# Mechanosensitive miR-99b mediates the regulatory effect of matrix stiffness on bone marrow mesenchymal stem cell fate both *in vitro* and *in vivo*

**DOI:** 10.1063/5.0131125

**Published:** 2023-01-18

**Authors:** Bojun Cao, Jiaxin Li, Xiaowen Wang, Zhaoyang Ran, Jia Tan, Liang Deng, Yongqiang Hao

**Affiliations:** 1 Shanghai Key Laboratory of Orthopaedic Implants, Department of Orthopaedic Surgery, Shanghai Ninth People's Hospital, Shanghai Jiao Tong University School of Medicine, Shanghai 200011, China; 2 Department of Orthopedics, The Second Affiliated Hospital of Harbin Medical University, Harbin, China; 3 Department of Cardiothoracic Surgery, The First Affiliated Hospital of Chongqing Medical University, Chongqing 400016, China; 4 Clinical and Translational Research Center for 3D Printing Technology, Shanghai Ninth People's Hospital, Shanghai Jiao Tong University School of Medicine, Shanghai 200011, China

## Abstract

Mechanical signals from extracellular matrix stiffness are important cues that regulate the proliferation and differentiation of bone marrow mesenchymal stem cells (BMSCs). However, the incorporation of BMSCs into soft hydrogels and the dominance of soft matrices for BMSC growth and differentiation limit the directed differentiation of BMSCs incorporated into hydrogels for tissue engineering, especially osteogenesis. Here, we found that the expression of miR-99b increased with increasing hydrogel stiffness and that miR-99b regulated the proliferation and differentiation of BMSCs seeded on the surface of substrates with different stiffnesses. Furthermore, miR-99b significantly promoted the migration of BMSCs in 3D hydrogels. Mechanistically, we demonstrated that matrix stiffness-sensitive miR-99b targets the mammalian target of the rapamycin signaling pathway to regulate the adipogenic and osteogenic differentiation of BMSCs. In addition, by modulating the expression of miR-99b, the osteogenic differentiation of BMSCs in soft 3D hydrogels was promoted. Consistently, the flexible BMSC-GelMA hydrogel transfected with miR-99b significantly promoted bone regeneration in the rat calvarial defect area. These results suggest that miR-99b plays a key role in the mechanotransduction and phenotypic transformation of BMSCs and may inspire new tissue engineering applications with MSCs as key components.

## I. INTRODUCTION

Bone marrow mesenchymal stem cells (BMSCs) are among the most promising and commonly used elements in tissue engineering and regenerative medicine due to their ability to differentiate into a variety of cell types, including osteoblasts, chondrocytes, and adipocytes.^1–5^ By combining BMSCs with biomaterials such as hydrogels and implementing appropriate MSC-based tissue engineering methods, we can repair and rebuild damaged tissues more efficiently.^6,7^ However, we can only effectively promote the formation of target tissue types by mastering the regulation of MSC differentiation in biomaterials to target phenotypes only.

Recent studies have shown that MSCs are highly sensitive to their surrounding microenvironment, including matrix surface morphology, viscoelasticity, and anisotropy.^8–11^ In particular, the effect of matrix stiffness on MSC proliferation and phenotypic differentiation has attracted increasing attention.^12–14^ Studies have shown that a stiff matrix promotes the osteogenic differentiation of MSCs, while a soft matrix promotes their differentiation to neurons or adipocytes.^15–18^ These results suggest that biomaterial properties significantly affect the differentiation of MSCs in tissue engineering materials.

Mcbeath *et al.*^17^ first reported the effect of mechanical cues on MCS differentiation. They demonstrated that MSCs that adhered, spread, and expanded showed osteogenic differentiation, while cells that were not fully spread and appeared round were more likely to differentiate into adipocytes. This cellular phenotype switch is mediated by a change in RhoA activity.

The regulation of mechanical cues serves as an effective method to guide the directional differentiation of MSCs.^19,20^ However, traditional regulatory methods, such as the use of small-molecule inhibitors or growth factors, lack stability and specificity to target cells, so their translational applications in clinical development are relatively limited. Therefore, the use of microRNA is a promising method of regulating mechanotransduction. miRNAs are a class of short noncoding RNA molecules that negatively regulate the expression of genes by inducing translational repression and mRNA degradation.^21,22^ Regulating cellular activity with miRNA is relatively simple and has relatively high regulation efficiency and low cost. miRNAs have been shown to play an important role in the proliferation and phenotypic differentiation of MSCs.^23–27^ In addition, studies have found that miRNAs regulate the interfacial behavior of MSCs cultured on microgrooved substrates. Through miRNA sequencing, Wang *et al.*^28^ found that the expression levels of miR-140, miR-210, miR-214, miR-351, and miR-99b were significantly increased in MSCs cultured on microgrooved substrates compared with MSCs cultured on smooth substrates. Several of these miRNA mimic combinations were transfected into MSCs cultured on smooth substrates, significantly increasing osteogenesis-related genes' expression. This suggests that miRNAs may play an important role in mechanosignaling and MSC differentiation. However, it is unclear whether these miRNAs are regulated by matrix stiffness; their possible regulatory mechanism is also unknown.

In this study, we found that the expression of miR-99b increased with increasing hydrogel matrix stiffness. In addition to affecting the proliferation and migration of BMSCs, the regulation of miR-99b expression could also affect the osteogenic or adipogenic differentiation of BMSCs by targeting the mTOR signaling pathway. Finally, we demonstrated that increased expression of miR-99b promotes the osteogenic differentiation of BMSCs in soft 3D hydrogels and promotes bone regeneration in rat calvarial defect areas. These results demonstrate that miR-99b plays a key role in the mechanotransduction and phenotypic transformation of BMSCs, suggesting its value in MSC-based tissue engineering applications.

## II. RESULTS

### A. Substrate stiffness affects the morphology and phenotypic differentiation of BMSCs

**FIG. 1.**
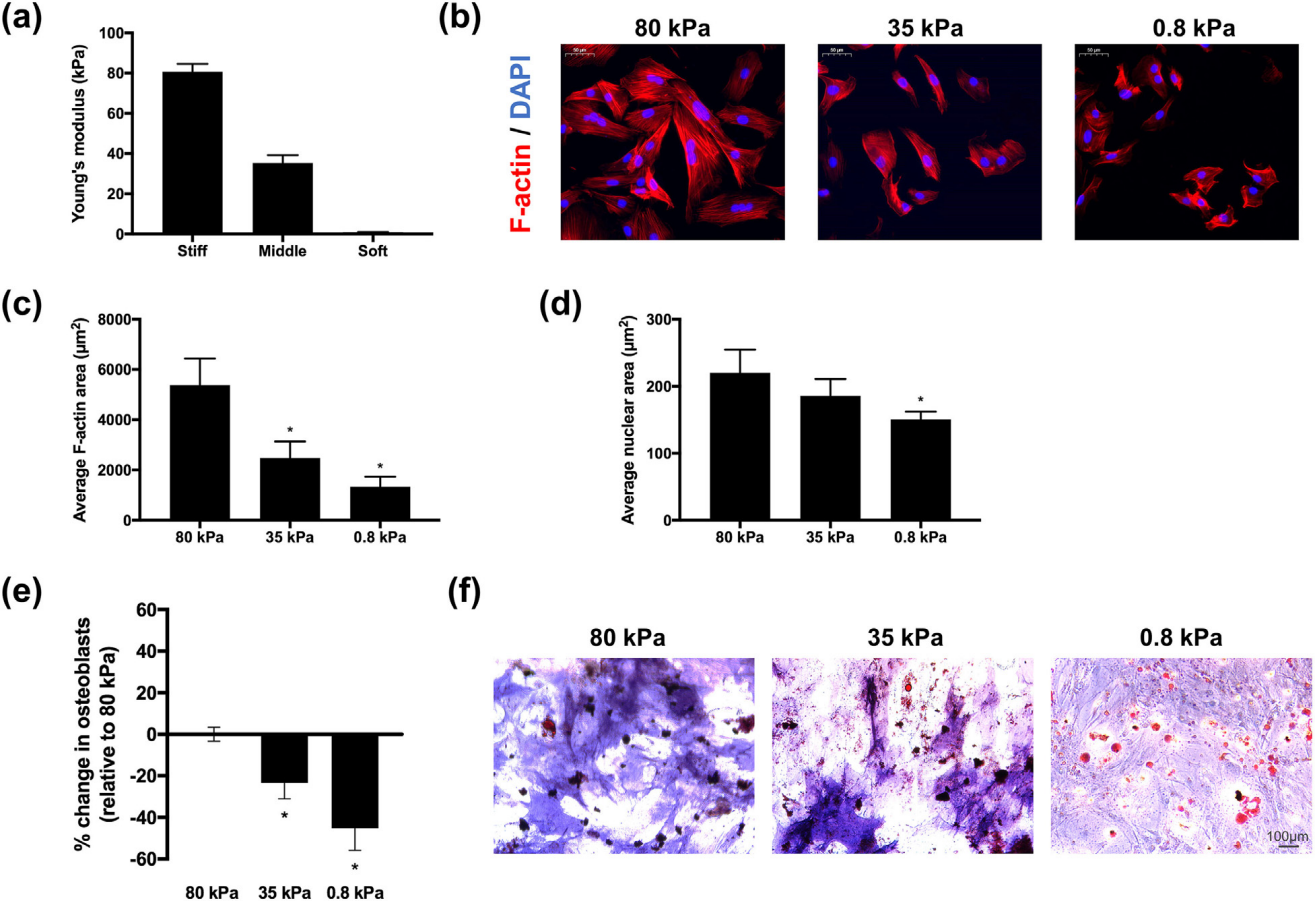
Effects of matrix stiffness on BMSC morphology and phenotypic differentiation. (a) Young's modulus of three groups of PA gels ranging from soft to stiff (n = 3). (b) Fluorescent staining of BMSCs cultured on gels of different stiffnesses showing actin (red) and nuclei (blue). Average area of actin filaments (c) and nuclei (d) of BMSCs cultured on gels with different stiffnesses (n = 4). (e) Differentiation bias of BMSCs cultured on gels of different stiffnesses. Data are shown as the mean ± SD of the change in the percentage of osteoblasts (n = 4). (f) Representative images of Oil Red O (red) and alkaline phosphatase (blue) staining of BMSCs cultured on gel surfaces with different stiffnesses. Differences between groups were analyzed by one-way ANOVA with Tukey's post-hoc test, ^*^p <0.05.

We prepared three groups of polyacrylamide (PA) gels ranging from soft to hard with Young's moduli of approximately 0.8, 35, and 80 kPa and applied 25 *μ*g/ml collagen I to the surface of the gels by cross-linking [Fig. 1(a)]. Actin fibers were stained with phalloidin after seeding BMSCs on gel surfaces of different stiffnesses for 24 h. The morphology of BMSCs showed marked differences, with BMSCs on relatively hard substrates (35 and 80 kPa) spread more widely with thick and aligned actin fibers, while BMSCs on soft substrates had a smaller spread area and randomly oriented actin fibers [Fig. 1(b)]. After measurement, it was found that the average areas of actin fibers and nuclei were 1339.25 and 150.65 *μ*m^2^ on the surface of the soft gel (0.8 kPa), respectively, and 5377.50 and 219.75 *μ*m^2^ on the surface of the hard gel (80 kPa) [Figs. 1(c) and 1(d)].

To clarify the effects of soft and hard matrices on the osteogenic and adipogenic differentiation of BMSCs, we used a mixture of osteogenic and adipogenic induction medium (1:1) to culture cells for 15 days and found that BMSCs on hard matrices mainly differentiated into osteoblasts. However, with decreasing matrix stiffness, BMSCs gradually tended toward adipogenic differentiation [Figs. 1(e) and 1(f)].

### B. miR-99b expression in response to changes in gel substrate stiffness

miRNAs have been shown to play a key role in the regulation of MSC fate, including proliferation, migration, and phenotypic differentiation. More interestingly, some miRNAs were found to regulate the interface behavior of MSCs cultured on microgrooved substrates. However, whether these miRNAs are regulated by matrix stiffness and their possible regulatory mechanisms are still unknown.

**FIG. 2.**
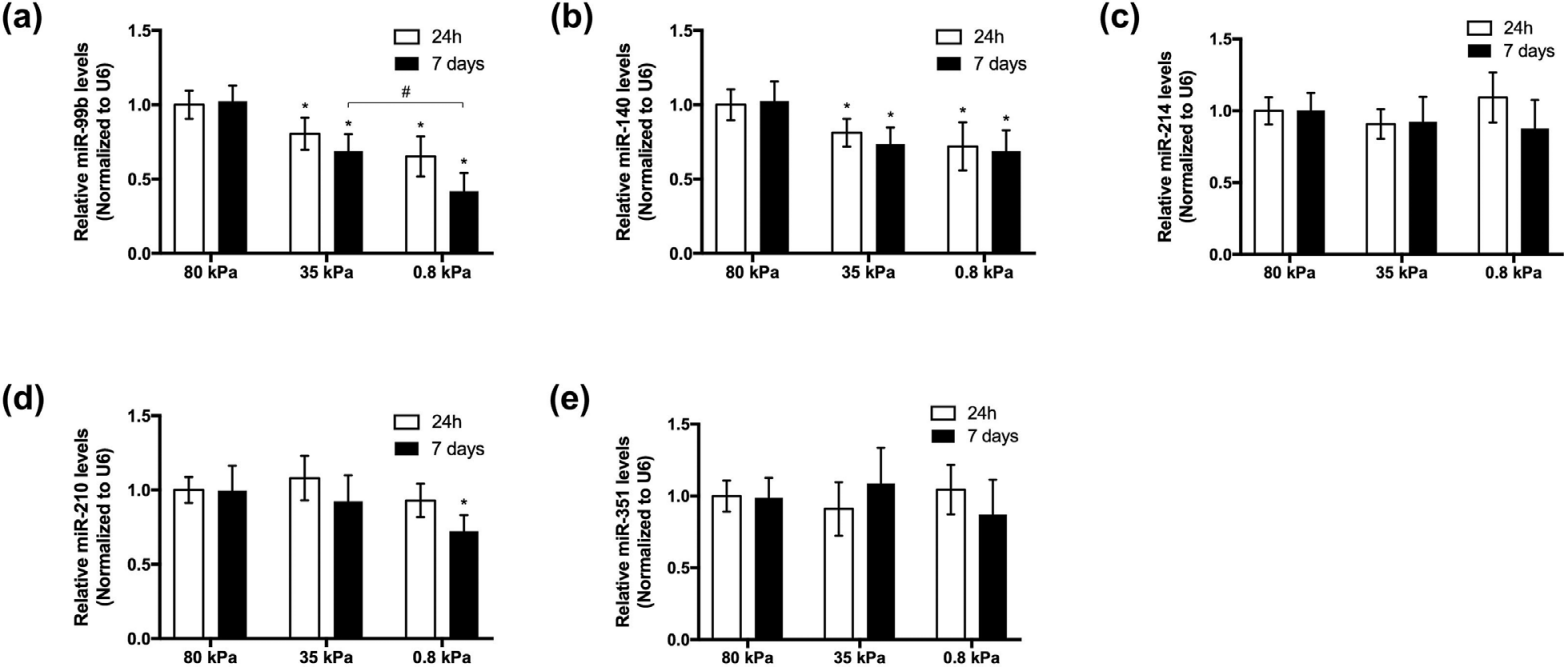
The expression of each miRNA was detected by qPCR after BMSCs were cultured on gels of different stiffnesses for 24 h (white) and 7 days (black). (a) Expression of miR-99b. (b) Expression of miR-140. (c) Expression of miR-214. (d) Expression of miR-210. (e) Expression of miR-351. n = 4. Differences between groups were analyzed by one-way analysis of variance (ANOVA) with Tukey's post-hoc test. ^*^P <0.05 compared with the 80 kPa group, #P <0.05.

Therefore, we cultured BMSCs on gel substrates with elastic moduli of 80, 35, and 0.8 kPa for 24 h and detected the levels of miR-140, miR-210, miR-214, miR-351, and miR-99b by qPCR [Figs. 2(a)–2(e)]. The results showed that the expression levels of miR-99b and miR-140 gradually decreased with decreasing gel substrate stiffness [Figs. 2(a) and 2(b)]. Furthermore, we extended the culture time of the cells to 7 days and found that the above-mentioned miRNA expression trends were largely maintained, and differences were more significant [Figs. 2(a) and 2(b)].

### C. Regulation of miR-99b can alter the phenotypic differentiation bias of BMSCs

Given the significant differences in the expression of miR-99b and miR-140 in response to changes in gel substrate stiffness, we next continued to investigate the effects of the regulation of these differentially expressed miRNAs on the osteogenic and adipogenic differentiation bias of BMSCs. BMSCs were seeded on the surface of both soft and hard gels, and a mixed induction medium was used. Then, cells were transfected with miRNA mimics or inhibitors, and the deviation of osteogenic and adipogenic differentiation of cells was detected after 15 days of culture.

**FIG. 3.**
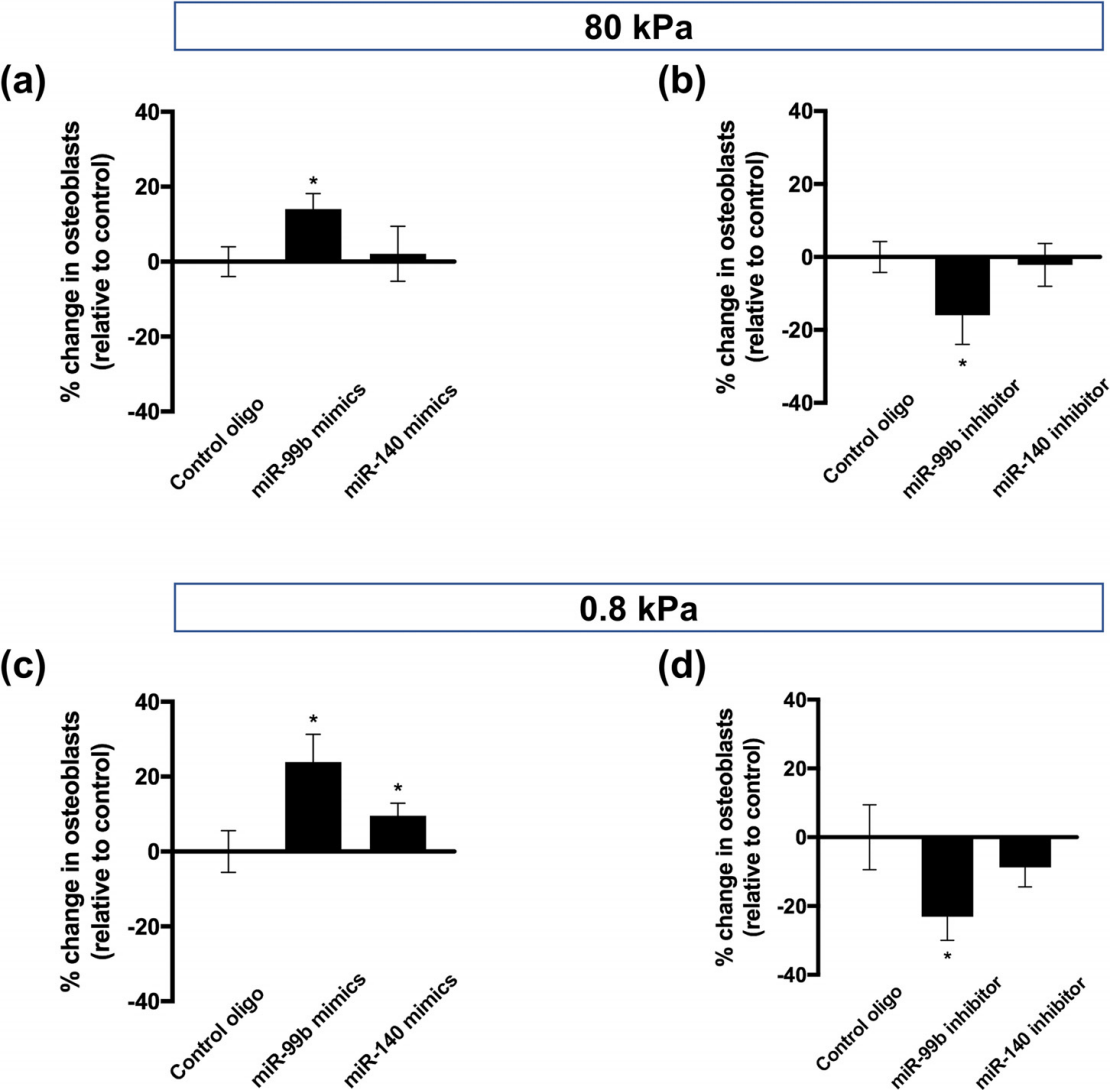
Regulation of miR-99b expression can modulate BMSC differentiation bias in response to matrix stiffness. Differentiation bias of BMSCs treated with (a) miRNA mimics or (b) miRNA inhibitors and cultured on 80 kPa gels. Differentiation bias of BMSCs treated and cultured with (c) miRNA mimics or (d) miRNA inhibitors on 0.8 kPa gels. Differences between groups were analyzed by one-way ANOVA with Tukey's post-hoc test, ^*^p <0.05.

On gels with matrix stiffnesses of 80 and 0.8 kPa, we found no consistent effect of miR-210 on the differentiation bias of BMSCs. However, miR-99b mimics significantly increased the proportion of osteoblasts compared with blank control or control oligonucleotide-treated cells [Figs. 3(a) and 3(c)], and inhibition of miR-99b expression had the opposite effect on the differentiation bias of BMSCs [Figs. 3(b) and 3(d)].

### D. Regulation of miR-99b can affect BMSC proliferation and migration in response to matrix stiffness

**FIG. 4.**
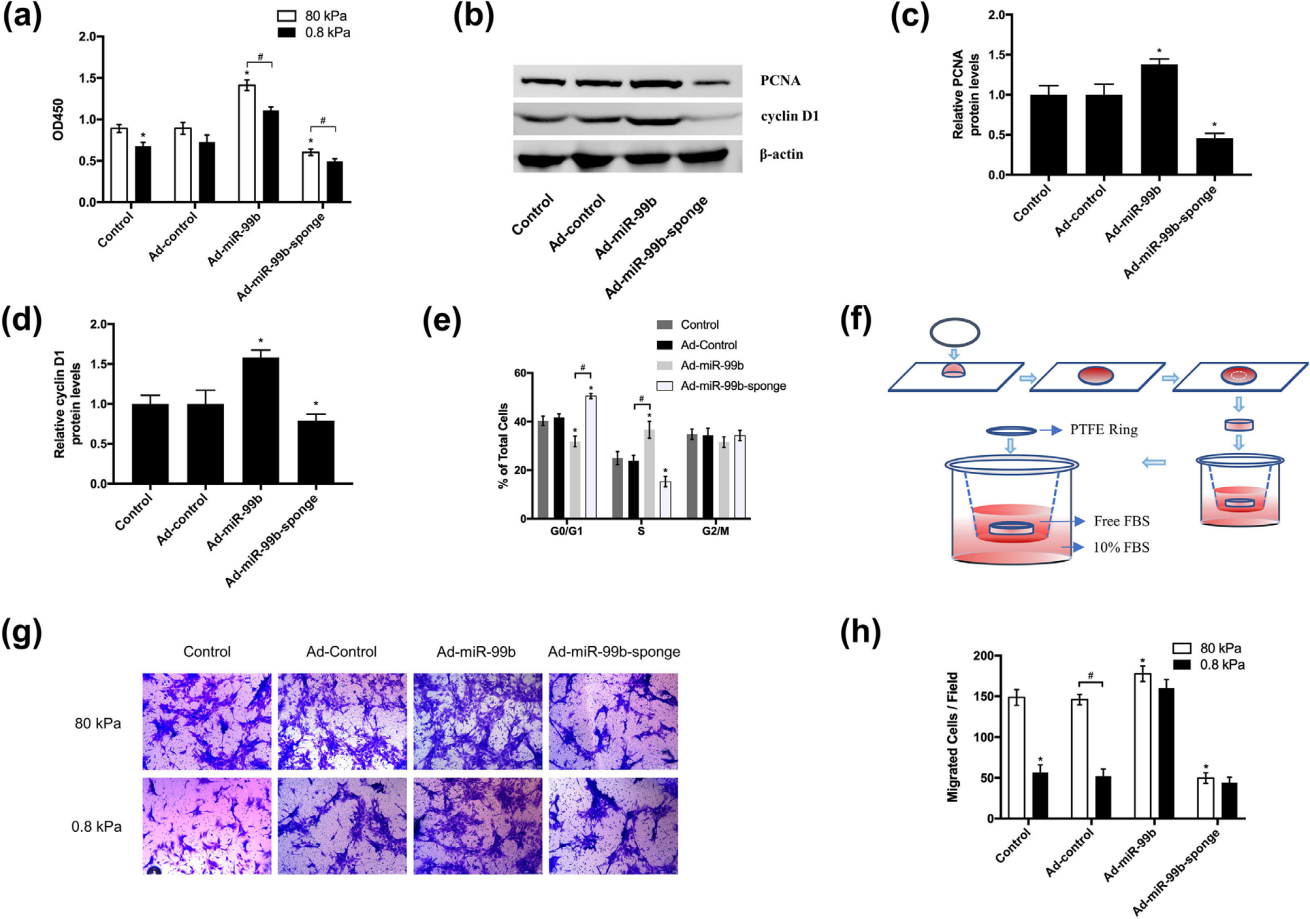
miR-99b promotes BMSC proliferation and migration. (a) The effect of miR-99b on BMSC proliferation on substrates of different stiffnesses was determined by CCK-8 (n = 4). (b)–(d) Western blot analysis showed that ad-miR-99b significantly promoted the expression of PCNA and cyclin D1 (n = 3). (e) The effect of miR-99b on BMSC cell cycle distribution was analyzed by flow cytometry (n = 3). (f) The effect of miR-99b on BMSC cell cycle distribution was analyzed by flow cytometry (n = 3). (g) and (h) miR-99b mediates the regulation of BMSC migration by matrix stiffness under 3D conditions (n = 4). ^*^p <0.05, #p <0.05.

By performing the CCK-8 assay, we found that a low stiffness gel matrix inhibited the proliferation of BMSCs while increasing the expression of miR-99b in BMSCs could significantly promote cell proliferation [Fig. 4(a)]. Western blot results showed that the overexpression of miR-99b significantly increased the expression of PCNA and cyclin D1, which are crucial regulators of G1 to S phase progression in many cell types [Figs. 4(b)–4(d)]. Consistently, by cell cycle analysis, we found that Ad-miR-99b-sponge promoted the accumulation of cells in the G0/G1 phase and a lower proportion of cells in the S phase [Fig. 4(e)].

We then used transwell analysis to investigate whether miR-99b could mediate the regulation of matrix stiffness on BMSC migration under 3D conditions. First, BMSCs were transfected with Ad-control, Ad-miR-99b, or Ad-miR-99b-sponge, the BMSC-encapsulated hydrogels with different stiffnesses were cut into disks, and the gel disks were placed in transwells [Fig. 4(f)]. BMSC migration was induced by FBS in the lower chamber. Cell staining and counting showed that compared with the results in the high-stiffness gel matrix, the migration ability of cells in the low-stiffness gel matrix was significantly decreased, while overexpression of miR-99b could significantly promote the migration of BMSCs [Figs. 4(g) and 4(h)].

### E. Matrix stiffness-sensitive miR-99b targets mTOR to regulate BMSC differentiation

**FIG. 5.**
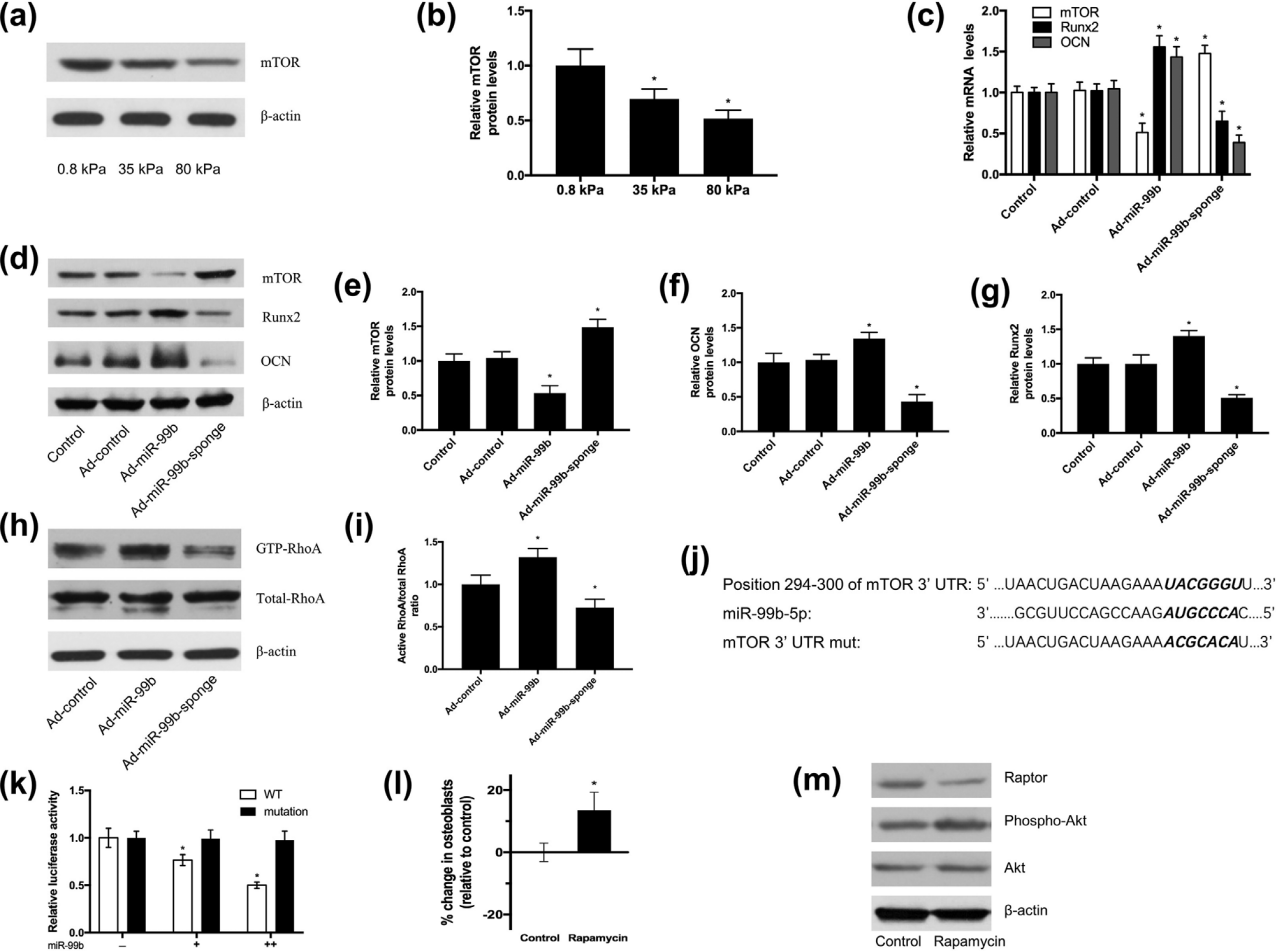
miR-99b targets mTOR to regulate BMSC differentiation. (a) Western blot analysis of mTOR in response to matrix stiffness. (b) Quantitative analysis of the protein expression shown in (a) was normalized to β-actin (n = 3). (c) miR-99b markedly reduced the expression of mTOR and promoted the expression of the osteogenesis-related genes OCN and Runx2 in BMSCs, as determined by qRT–PCR (n = 3). (d) Western blot analysis showed that ad-miR-99b significantly reduced the expression of mTOR and promoted the expression of osteogenesis-related genes OCN and Runx2 in BMSCs, while ad-miR-99b-sponge had the opposite effect. (e)–(g) Quantitative analysis of the protein expression shown in (d) normalized to β-actin (n = 3). (h)–(i) miR-99b significantly enhanced the activity of RhoA. (j) The predicted miR-99b binding site within the mTOR 3′-UTR by TargetScan. (k) Luciferase reporters including the mTOR 3′-UTR harboring the wild-type or mutated miR-99b binding site were constructed, and transfection experiments were performed. Reporter activity was normalized to the Renilla luciferase internal control and expressed relative to the empty plasmid transfection. (l) Differentiation bias analysis of BMSCs treated with 100 nM rapamycin. (m) Effects of treatment with 100 nM rapamycin on the mTOR signaling pathway of BMSCs, as determined by Western blot analysis. Differences between groups were analyzed by one-way ANOVA with Tukey's post-hoc test, ^*^p <0.05.

Next, we verified that both the mRNA and protein expression levels of Runx2 and osteocalcin (OCN) in BMSCs transfected with Ad-pri-miR-99b were significantly increased. In contrast, Ad-miR-99b-sponge significantly decreased the expression of Runx2 and OCN [Figs. 5(c)–5(g)]. Furthermore, we found that increasing the expression of miR-99b significantly enhanced RhoA activity [Fig. 5(h) and 5(i)]. Studies have reported that the mTOR signaling pathway is involved in regulating the osteogenic or adipogenic differentiation of MSCs. We found that the expression of mTOR decreased with increasing matrix stiffness [Figs. 5(a) and 5(b)] and that increasing the expression of miR-99b significantly reduced the expression of mTOR mRNA and protein, while ad-miR-99b-sponge significantly increased the expression of mTOR mRNA and protein [Figs. 5(c)–5(e)]. Combined with TargetScan's algorithm to predict the binding site of the mTOR gene 3′-UTR and miR-99b, we preliminarily concluded that mTOR is a potential target of miR-99b [Fig. 5(j)].

To experimentally validate mTOR as a miR-99b target gene, we first constructed luciferase reporters including the mTOR 3′-UTR harboring the wild-type or mutated miR-99b binding site, and then performed transfection experiments. Experimental data showed that the luciferase activity of the wild-type mTOR 3′-UTR luciferase reporter was markedly reduced with increasing doses of miR-99b mimic, whereas no significant changes were observed in the luciferase activity of the mutant constructs, suggesting that miR-99b directly downregulates mTOR by its 3′-UTR binding site [Fig. 5(k)].

We then applied an inhibitor of mTOR, rapamycin, to BMSCs to determine whether it has a similar effect to miR-99b in targeting and downregulating mTOR expression. The results of phenotypic differentiation bias experiments on BMSCs showed that rapamycin had a similar effect to miR-99b mimics and significantly promoted the osteogenic differentiation of cells [Fig. 5(l)]. Western blotting further demonstrated that rapamycin inhibits the mTORC1 pathway and induces Akt phosphorylation [Fig. 5(m)]. These results are consistent with previous studies showing that MSCs from mTORC1 knockout mice tend to differentiate into osteogenic cells and that rapamycin promotes osteogenesis.

**FIG. 6.**
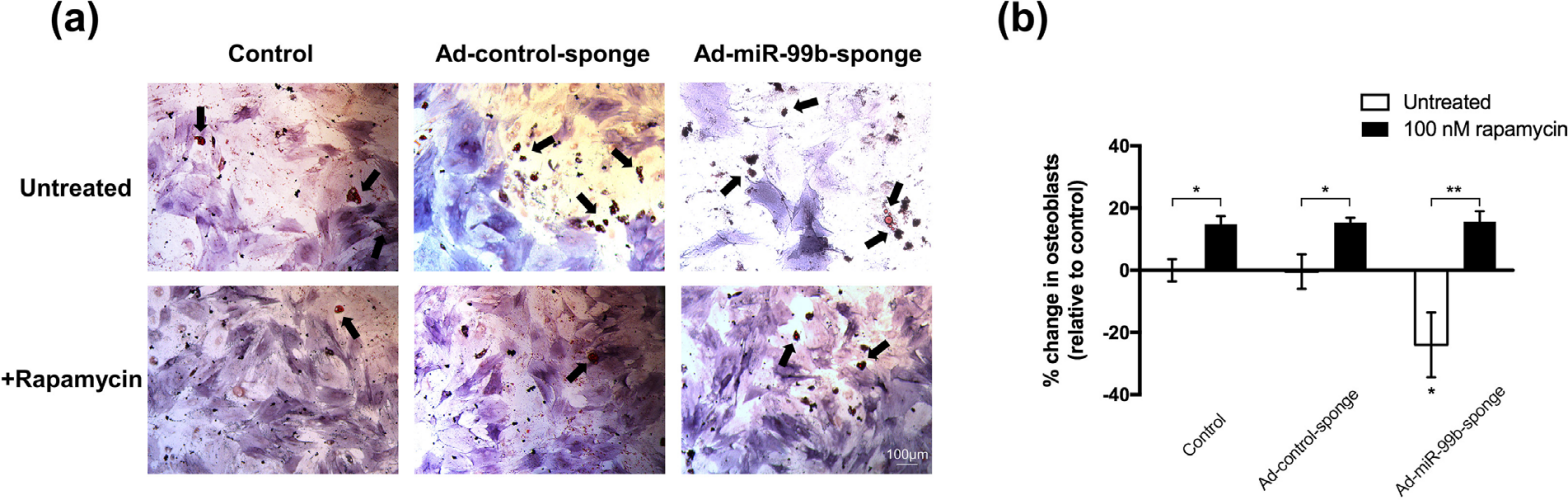
Rapamycin surpassed the effect of Ad-miR-99b-sponge and significantly promoted the osteogenic differentiation of BMSCs. (a) Representative images of Oil Red O (red) and alkaline phosphatase staining of Ad-miR-99b-sponge-transfected BMSCs with and without 100 nM rapamycin treatment. (b) Osteogenic and adipogenic differentiation bias of Ad-miR-99b-sponge-transfected BMSCs with and without 100 nM rapamycin treatment. Data are shown as the mean ± SD of the change in the percentage of osteoblasts (n = 4). Differences between groups were analyzed by one-way ANOVA with Tukey's post-hoc test, ^*^p <0.05, ^**^p <0.01.

Next, to further verify that mTOR signaling pathway transduction is regulated by miR-99b, BMSCs were transfected with Ad-miR-99b-sponge and treated with 100 nM rapamycin. The inhibition of miR-99b expression promoted the adipogenic differentiation bias of BMSCs, resulting in a significant decrease in the proportion of osteoblasts, which was consistent with our previous findings. However, the addition of 100 nM rapamycin significantly enhanced the osteogenic differentiation of BMSCs, and at the same time, no significant difference was observed between the groups treated with rapamycin, demonstrating that the inhibition of mTOR signaling is sufficient to overcome the cell phenotypic differentiation-biased differences induced by ad-miR-99b-sponge [Figs. 6(a) and 6(b)]. The above results further verify that miR-99b affects the differentiation fate of BMSCs by targeting mTOR.

### F. Regulation of miR-99b expression can promote the differentiation of BMSCs in soft 3D hydrogels

We then verified whether modulation of miR-99b expression could affect the differentiation of BMSCs in soft 3D GelMA hydrogel structures (elastic modulus ∼3.2 kPa, supplementary material Fig. 1). Briefly, BMSCs were transfected with ad-pri-miR-99b, encapsulated in photocrosslinkable GelMA hydrogels and then cultured with osteogenic induction medium for 15 days.

**FIG. 7.**
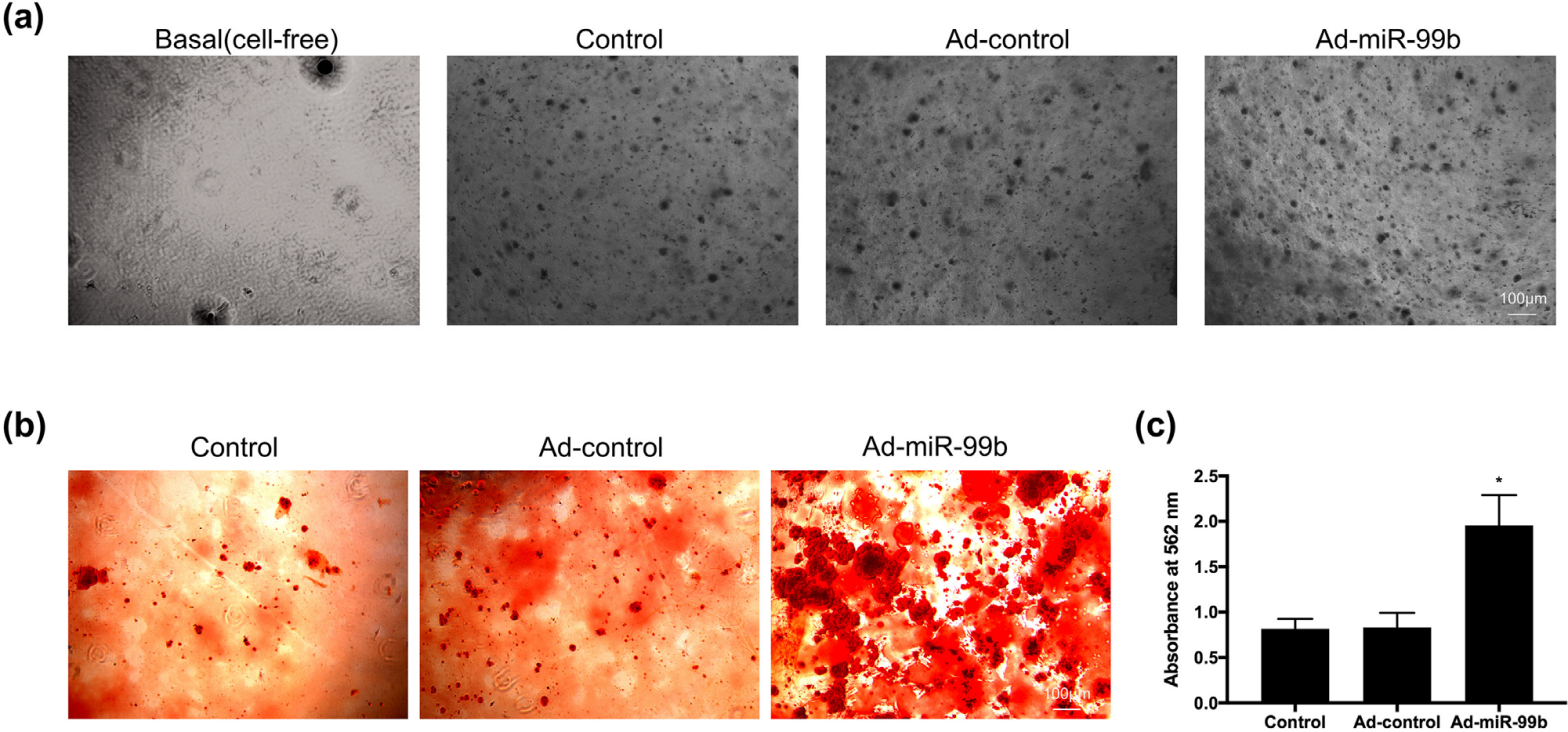
miR-99b can significantly promote the osteogenic differentiation of BMSCs in 3D hydrogels. (a) Representative images of BMSCs within 3D hydrogels 15 days after osteogenic induction. (b) Alizarin red staining after 15 days of osteogenic induction of BMSCs within 3D hydrogels. (c) Quantitative analysis of Alizarin red S staining. n = 5, differences between groups were analyzed by one-way ANOVA with Tukey's post-hoc test, ^*^p <0.05.

After 15 days of culture, through microscopy, we found that numerous BMSCs in the hydrogel agglomerated and turned black and granular [Fig. 7(a)]. Macroscopic observation showed that with the extension of the culture period, the hydrogel gradually became opaque, and white deposits were visible to the naked eye after the fixation of some of the hydrogel samples, which we deduced to be calcium salt deposits.

Afterward, the samples of each group were observed by alizarin red staining, treated with cetyl pyridine chloride reagent, and quantitatively analyzed by means of a microplate reader. The results showed that compared with the control group, the amount of calcium salt deposition in the Ad-pri-miR-99b treatment group was significantly increased [Figs. 7(b) and 7(c)].

### G. Ad-miR-99b-BMSC-GelMA enhances rat calvarial regeneration

**FIG. 8.**
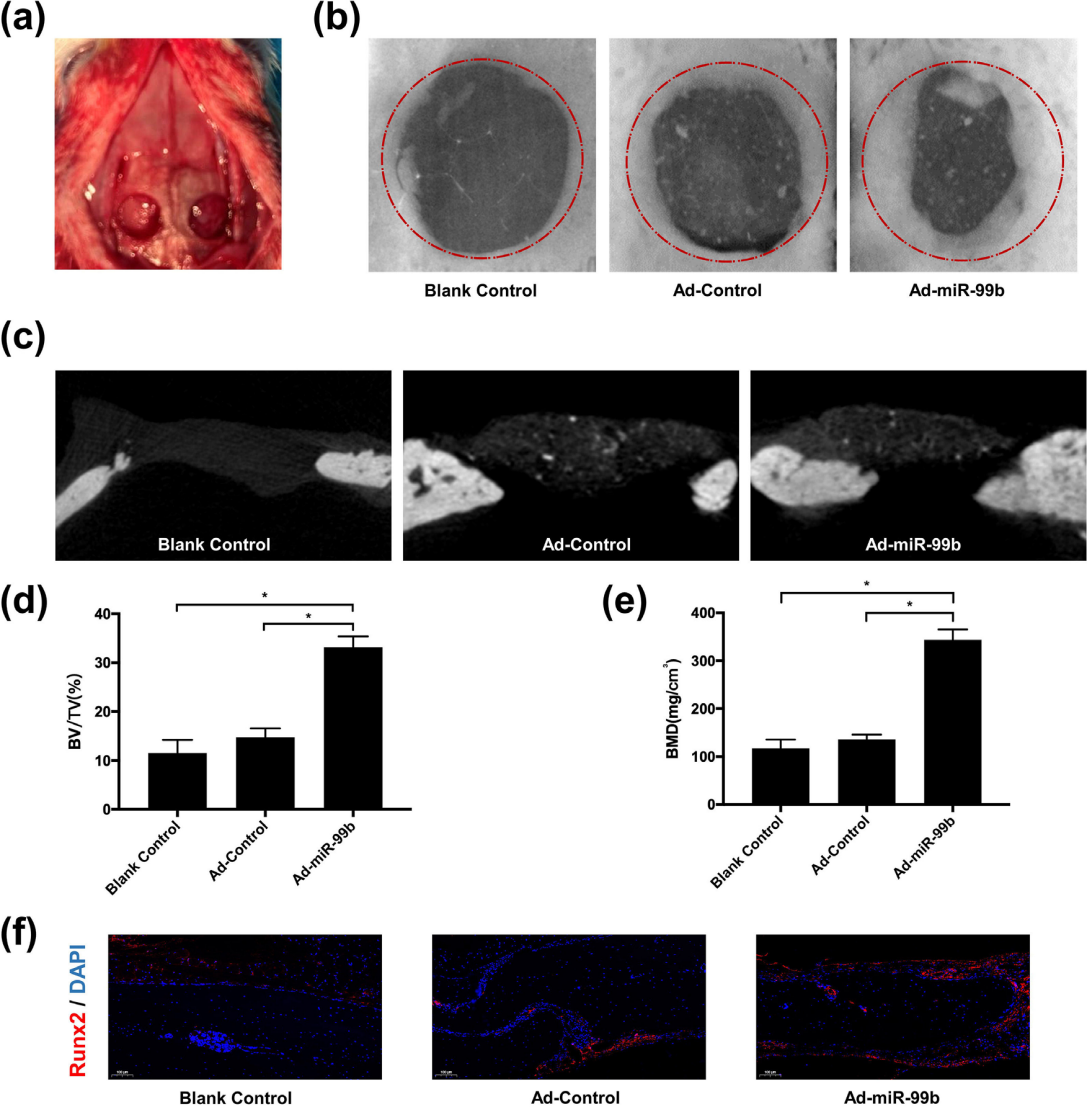
Bone regeneration after Ad-miR-99b-BMSC-GelMA implantation. (a) Construction of the rat calvarial defect model. (b) Three-dimensional micro-CT reconstruction of the calvarial defect at 6 weeks after implantation. (c) Cross section of the calvarial defect area. (d)–(e) Quantification of bone mineral density (BMD) and bone volume to total bone volume (BV/TV). (f) Representative immunofluorescence staining of Runx2 in skull tissue sections. n = 8, ^*^p <0.05.

Finally, we constructed a rat skull defect model to test the bone regeneration ability of the Ad-miR-99b-BMSC-GelMA flexible hydrogel scaffold implanted *in vivo* [Fig. 8(a)]. Micro-CT scanning and three-dimensional reconstruction imaging were performed on the sixth week after the operation. The reconstructed images showed that the new bone tissue in the Ad-miR-99b-BMSC-GelMA treatment group was significantly greater than that in the Ad-control-BMSC-GelMA control group and the blank control group [Figs. 8(b) and 8(c)]. Bone mineral density (BMD) and bone volume to total bone volume (BV/TV) were quantitatively calculated by CTAn software. The results showed that the bone volume fraction (BV/TV) and BMD values of the Ad-miR-99b-BMSC-GelMA treatment group were significantly higher than those of the control group [Figs. 8(d) and 8(e)]. In addition, we performed immunofluorescence staining for Runx2 in the area of the skull defect, and the results showed that the Ad-miR-99b-BMSC-GelMA-treated group expressed significantly more Runx2.

## III. DISCUSSION AND CONCLUSION

Bone marrow-derived mesenchymal stem cells have always been an important source of seed cells for tissue repair and regenerative medicine due to their multiple differentiation potentials. Therefore, it is particularly important to control the differentiation of MSCs.^29,30^ Studies have shown that the regulation of MSC differentiation is not only affected by biochemical factors but also closely related to the physical stimulation of their surrounding microenvironment. In particular, matrix stiffness has been widely shown to be an important factor affecting the proliferation and differentiation of MSCs, but here, we demonstrate for the first time that-mechanosignaling involving miR-99b plays an important role in regulating the response of rat BMSCs to the surrounding physical environment. Furthermore, regulating substrate stiffness-sensitive miR-99b can be used to guide the directed differentiation of BMSCs.

By miRNA sequencing, Wang *et al.* found that the expression levels of miR-140, miR-210, miR-214, miR-351, and miR-99b were significantly increased in MSCs cultured on microgrooved substrates compared with MSCs cultured on smooth substrates. Several of these miRNA mimic combinations were transfected into MSCs cultured on smooth substrates, and the expression of osteogenesis-related genes was significantly increased. However, they did not conduct in-depth research on the connection and mechanism of action. This attracted our interest, and we speculated that miRNAs might play an important role in mediating between mechanosignaling and MSC differentiation. Thus, we sought to further verify whether the above target miRNAs could affect MSC fate in response to changes in substrate stiffness.

We constructed three groups of hydrogel substrates with an increasing Young's modulus gradient and found by qRT-PCR that the expression of miR-99b increased with increasing hydrogel stiffness. To make substrates with different stiffnesses, we chose PA gels with good biocompatibility and controllable surface stiffness.^9,20,31,32^ By adjusting the ratio of acrylamide and bis-acrylamide, the gel stiffness can be controlled to within several 100 Pa. The experiment had good repeatability up to 100 kPa and was able to simulate the stiffness of different tissues of the body. However, because the PA glue is prepared on the surface of the cover glass, the stiffness of the cover glass may have an impact on the surface stiffness of the PA glue. When the PA glue base is too thin, cells may pass through the PA glue base and sense the stiffness of the coverslip, affecting the experimental results. Therefore, to exclude this effect, the PA glue base must be thick enough to completely exclude the effect of the stiffness of the coverslip. Tian *et al.*^32^ found that the differences in gene expression in smooth muscle cells cultured on PA gels with different thicknesses were affected by the stiffness of the coverslip, while the differences in gene expression in SMCs decreased significantly with increasing substrate thickness. When the thickness was greater than 75 *μ*m, there was no significant difference in gene expression in SMCs, indicating that when the thickness of the substrate was greater than 75 *μ*m, SMCs could no longer sense the stiffness of the coverslip, and the difference in gene expression in SMCs arose from the stiffness of the PA glue. Based on this, we selected a PA glue substrate with a thickness of approximately 100 *μ*m for subsequent experiments.

We then used adenoviral vector tools to amplify and inhibit the expression of miR-99b in rat BMSCs cultured on soft and hard substrates, and we found that overexpression of miR-99b promoted the osteogenic differentiation of BMSCs. Through analysis of the miRNA target cell database, we preliminarily proposed mTOR as a potential target of miR-99b because the mTOR signaling pathway is known for regulating cell fate, and miR-99 has been reported to be associated with mTOR in previous studies and has been shown to affect cell proliferation, migration, and phenotypic differentiation by targeting mTOR, especially in tumor cells.^33–35^ In addition, we noticed that the two complexes of mTOR can affect cell differentiation by regulating the expression of osteogenic and adipogenic differentiation-related marker proteins and can regulate RhoA to affect cell activity.^36–38^ This was consistent with our experimental results, confirming that miR-99b targets mTOR to regulate mechanotransduction and differentiation of rat BMSCs.

Next, we demonstrated that treating BMSCs with low concentrations of rapamycin promoted osteogenic differentiation, which is consistent with the study by Gharibi *et al.*^39^ In addition, it is consistent with the conclusion of Martin *et al.*^40^ that MSCs from mTORC1 knockout mice showed reduced osteogenic differentiation ability.

The ability of rapamycin to override the action of the miR-99b sponge provides further evidence that the effect of basal stiffness on BMSC differentiation is mediated through the mTOR signaling pathway, and we demonstrate that miR-99b targets mTOR itself and, thus, may affect signaling through two complexes, mTORC1 and mTORC2. Our study found decreased expression of raptor and increased AKT-Ser473 phosphorylation in rapamycin-treated BMSCs, which agreed with the findings of Chen *et al.*^41^

Based on the above findings, we further demonstrated that when the expression of miR-99b in BMSCs encapsulated in 3D GelMA hydrogels was upregulated, calcium deposition was significantly increased, suggesting that the regulation of miR-99b promotes the generation of osteoblast populations. Finally, we constructed a rat calvarial defect model and demonstrated the bone regeneration ability of the Ad-miR-99b-BMSC-GelMA flexible hydrogel scaffold. Therefore, combining MSCs with biomaterials to use this newly discovered regulatory technique in clinical applications may help to promote the differentiation of MSCs in the desired direction.

**FIG. 9.**
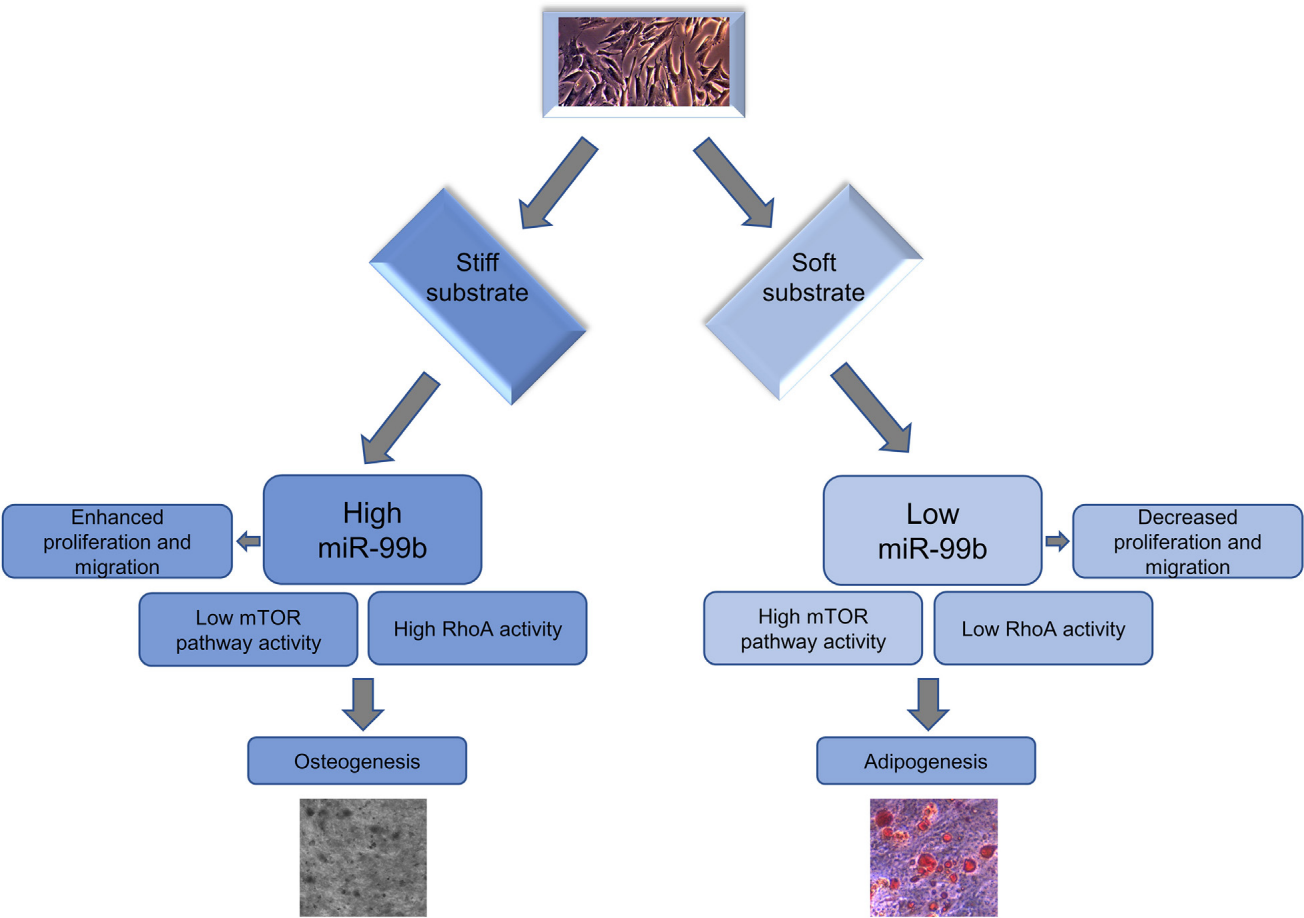
Simplified diagram of the overall research results.

In conclusion, we found for the first time that miR-99b expression responds to changes in hydrogel matrix stiffness and that the regulation of mechanosensitive miR-99b can affect the differentiation of BMSCs by targeting the mTOR signaling pathway. Furthermore, amplified expression of miR-99b can promote the osteogenic differentiation of BMSCs in soft 3D hydrogels. Consistently, the BMSC-GelMA flexible hydrogel transfected with miR-99b significantly promoted bone regeneration in the rat calvarial defect area (Fig. 9). These results suggest that miRNAs play a key role in the mechanotransduction and differentiation of BMSCs, suggesting their significant value in MSC-based tissue engineering and regenerative medicine applications.

## IV. METHODS

### A. Primary culture of rat BMSCs

The bilateral femurs of three-week-old male SD rats were quickly removed in a sterile environment, taking care to keep the marrow cavity closed. Then, ophthalmic scissors were used to cut the bone marrow cavity at both ends on the ultraclean bench, and the marrow cavity was rinsed four to six times with α-MEM containing 10% fetal bovine serum into a 10 ml petri dish using a sterile syringe. Then, the cells were placed in an incubator for 36–48 h of static culture, the nonadherent cells were removed, and the medium was replaced. When the cells grew to 85%–90% confluence, they were passaged, and the third, or fourth generation was used for subsequent experiments.

### B. Preparation of substrates with different stiffnesses

In this experiment, polyacrylamide (PA) hydrogel was used to make substrates with different stiffnesses. The relative concentrations of acrylamide, bisacrylamide, and other components were changed to adjust the stiffness of the hydrogel substrate. The specific ratio configurations are shown in Table I. Then, 0.5 *μ*l of TEMED and 5 *μ*l of 10% w/v ammonium persulfate were added per 500 *μ*l of the prepared master mix, and the solution was shaken slowly, taking care to avoid bubbles. The polymerization reaction was carried out for approximately 5 min, and after the solution was completely solidified, a PA-based adhesive with a thickness of approximately 100 *μ*m was prepared. The surface of the hydrogel was covered with 1 ml of 0.5 mg/ml sulfo SANPAH and then placed under a 365 nm UV lamp for approximately 10 min. After washing with PBS, 25 *μ*g/ml collagen I solution was coated on the hydrogel. The plates were allowed to stand for approximately 5 h at room temperature away from light. After sterilization, the gel was used for cell seeding.

**TABLE I.** Composition of PA gels with different stiffnesses.

	0.8 kPa	35 kPa	80 kPa
40% acrylamide (*μ*l)	75	184	246
2% bis-acrylamide (*μ*l)	10	70	180.5
1 M HEPES buffer (4-(2-Hydroxyethyl)-1-piperazineethanesulfonic acid) (*μ*l)	5	5	5
ddH_2_O (*μ*l)	410	241	68.5
Total (*μ*l)	500	500	500

### C. Measurement of the stiffness of PA hydrogels

Young's modulus of each group of hydrogel substrates was measured using atomic force microscopy. Briefly, the hydrogel substrate sample was immersed in PBS solution, 12 points on the sample were randomly selected for measurement, and each sample was randomly measured three times. Then, the data were collected for statistical analysis.

### D. Osteogenic and adipogenic induction and staining of BMSCs

BMSCs were seeded on PA gels with different stiffnesses at a density of 8000 cells/cm^2^, cultured in α-MEM containing 10% FBS until the cell density reached approximately 70%, and then switched to mixed osteogenic and adipogenic induction culture (1:1 volume ratio of osteogenic and adipogenic medium), and the medium was changed every 3 days. The osteogenic induction medium was primarily DMEM with 100 ng/ml dexamethasone, 10 mM β-glycerophosphate, 50 *μ*M ascorbic acid-2-phosphate, and 10% FBS. The adipogenic induction medium was primarily DMEM with 1 *μ*g/ml dexamethasone, 0.2 mM indomethacin, 10 *μ*g/ml insulin, 0.5 mM isobutyl-1-methylxanthine, and 10% FBS.

Alkaline phosphatase (ALP) and Oil Red O staining were performed on the 15th day of culture. Briefly, cells were gently washed three times with PBS, fixed with 4% paraformaldehyde at room temperature for 20 min, and then stained for 10 min in the dark using an ALP detection kit (Beyotime, Cat# P0321S). Next, the cells were stained with Oil Red O dye for 30 min in the dark and finally imaged with a microscope. we calculated the percentage of osteoblasts as the area of ALP-stained positive cells as a proportion of the total area of the images taken. The percentage of osteoblasts in the other stiffness groups minus the percentage of osteoblasts in the 80 kPa group was then obtained as “percentage change in osteoblasts (relative to 80 kPa).”

### E. Quantitative real-time PCR (qRT-PCR) analysis

qRT-PCR was performed as previously described, and total RNA from rat BMSCs was isolated with TRIzol reagent (Invitrogen). PCR analysis of miRNAs was performed using the following primers: miR-99b-5p (forward: ACACTCCAGCTGGGCACCCGTAGAACCGAC; reverse: TGGTGTCGTGGAGTCG), miR-140 (forward: ACACTCCAGCTGGGTACCACAGGGTAGAA; reverse: TGGTGTCGTGGAGTCG), miR-214 (forward: ACACTCCAGCTGGGAGAGTTGTCAT; reverse: TGGTGTCGTGGAGTCG), miR-210 (forward: ACACTCCAGCTGGGCTGTGCGTGTGACAGC; reverse: TGGTGTCGTGGAGTCG), miR-351 (forward: ACACTCCAGCTGGGTCCCTGAGGAGCCCTTTGA; reverse: TGGTGTCGTGGAGTCG), and U6 (forward: CTCGCTTCGGCAGCACA; reverse: AACGCTTCACGAATTTGCGT). The expression level of U6 was used as an endogenous control.

### F. Western blot analysis

Proteins were isolated from rat BMSCs, and Western blotting was performed as described previously. Antibodies against β-actin (Abcam), mTOR (Abcam), OCN (Abcam), Runx2 (Abcam), proliferating cell nuclear antigen (PCNA) (Abcam), cyclin D1 (Abcam), AKT (cell signaling), phosphorylated AKT (cell signaling), RhoA (Abcam), and raptor (Abcam) were used for immunoblotting. Images were captured by an ImageQuant LAS4000 Imaging Station (GE), and band densities were quantified by using ImageQuant TL software (GE).

### G. Cell proliferation assay

Cell proliferation was detected using a Cell Counting Kit-8 (CCK-8) assay (Beyotime Biotechnology, China). Briefly, BMSCs were seeded in 96-well plates at 10 000 cells per well and were transfected with Ad-control, Ad-miR-99b, and Ad-miR-99b-sponge, and the proliferation assay was performed when the cell growth density reached 70%.

### H. Cell cycle analysis

Cell cycle analysis using flow cytometry. Cells were transfected with Ad-control or Ad-miR-99b-sponge and cultured for 48 h, harvested, fixed with prechilled 75% ethanol at 4 °C, and then stained with the propidium iodide reagent. Finally, the cell cycle distribution was analyzed by FACScan.

### I. Cell migration assay

Cell migration ability was detected using a 24-well Transwell chamber (Corning, pore size: 8 *μ*m). Briefly, BMSCs transfected with Ad-control, Ad-miR-99b, or Ad-miR-99b-sponge were mixed with the gel to make gel disks with different elastic modulus sizes. Then, the gel disks were placed in the upper chamber, and 200 *μ*l of 0% FBS medium was added using an inner plastic ring (Teflon ring) to prevent the gel dish from floating. The number of cells in the gel dish in the upper chamber was approximately 15 000. For the lower chamber, 600 *μ*l of 10% the FBS medium was added to induce migration. After 36 h of incubation, cells on the underside of the membrane were fixed with 4% paraformaldehyde and subsequently stained with crystal violet solution. Images were taken, and the cells were counted.

### J. Dual luciferase reporter assay

To construct the mTOR 3′-untranslated region (3′-UTR) reporter, a fragment of the mTOR 3′-UTR including a putative miR-99b binding site was amplified by PCR with primers containing Pme I and Sac I restriction enzyme sites and then inserted into the 3′ end of the firefly luciferase gene in the pMir-report vector (Ambion, Applied Biosystems). A mutation of the miR-99b seeding site was introduced into the pMir-report by a QuickChange Site-Directed Mutagenesis Kit (Agilent Technologies) according to the manufacturer's instructions. The level of luciferase activity was then assessed by measuring the activity of firefly luciferase relative to that of the control (TKrenilla) according to the manufacturer's instructions.

### K. Construction of the miR-99b and miR-99b-sponge adenoviral vectors

Adenoviruses expressing miR-99b (Ad-miR-99b), miR-99b-sponge (Ad-miR-99b-sponge), and control viruses expressing GFP (Ad-control) were made using pAdEasy-EF1-MCS-CMV-EGFP (Hanbio Biotechnology) adenovirus vectors. The primer sequences of ad-miR-99b were as follows: Adeasy-rno-miR-99b-F: ctgtgaccggcgcctactctggtaccggagggctgggatgaggacccct, Adeasy-rno-miR-99b-R: atcttatctagaagcttaggctcgagctcctcgggtgtcttcctcaact; Adeasy-rno-miR-99b-5p-sponge-f: TGacatcCGCAAGGTCGGAATACGGGTGtcttcaCGCAAGGTCGGAATA, and Adeasy-rno-miR-99b-5p-sponge-r: TTCCGACCTTGCGgatgtCACCCGTATTCCGACCTTGCGgtataCACCCGTATTC. The test result of the adenovirus titer was approximately 5.0 × 10^10^ plaque forming units (pfu)/ml, as measured by a plaque titration assay in 293T cells.

### L. Alizarin red quantitative analysis

For this analysis, 500 *μ*l of de-ionized water was added to cover each well of a 24-well culture plate and stored in a 4 °C refrigerator in the dark for approximately one week to remove nonspecific staining. Then, 400 *μ*l of 10% cetylpyridine was added to each well, and the plates were protected from light for approximately 30 min at room temperature. One hundred microliters of cetylpyridine were transferred to each well of a 96-well plate, and the absorbance was measured with a microplate reader at 562 nm.

### M. Phalloidin staining of BMSCs

The cell-seeded slides were washed with PBS, fixed with 4% paraformaldehyde in the dark for 20 min at room temperature, washed again, incubated with diluted phalloidin staining solution (5 *μ*g/ml) for 1 h, and washed. After cleaning, DAPI was added dropwise and incubated in the dark for 5 min. After washing, images were taken under a fluorescence microscope.

### N. 3D GelMA hydrogel

A 5% (w/v) concentration of GelMA (EFL-GM-30–5%) was used to encapsulate BMSCs, and its elastic modulus was approximately 3.2 kPa, as measured by a nanoindenter. BMSCs were transfected with ad-miR-99b for 48 h before encapsulation in 3D hydrogels, mixed into the gel at a concentration of 1 × 10^6^/ml, and cultured in an osteogenic induction medium for 15 days. The culture medium was changed every 3 days.

### O. Rat calvarial defect repair operation

Male Sprague–Dawley rats (250–300 g) were fully anesthetized using an electric trephine to create bilateral 5 mm calvarial defects. Ad-control-BMSC-GelMA gel was used as the control group, and Ad-miR-99b-BMSC-GelMA gel was used as the experimental group. Animal sampling was performed after 6 weeks.

All protocols involving experimental animals were approved by the Animal Welfare Ethics Committee of the Ninth People's Hospital Affiliated to Shanghai Jiaotong University School of Medicine (SH9H-2019-A668–1), and all experimental procedures complied with the guidelines for the care and use of laboratory animals.

### P. Statistical analysis

Data are expressed as the mean ± SD, and statistical analysis was performed using GraphPad Prism 8.0. One-way ANOVA followed by Tukey's multiple comparison tests was applied for comparisons between multiple groups, and Student's t tests were applied for comparing differences between two groups. Kruskal–Wallis tests were used to analyze nonparametric data. P <0.05 was considered statistically significant.

## SUPPLEMENTARY MATERIAL

See the supplementary material for additional figures (supplementary material Fig. 1), which provide data for the elastic modulus of GelMA hydrogels with different concentrations and types.

## Data Availability

The data that support the findings of this study are available within the article and its supplementary material.
